# A Rare, Recurrent, *De Novo* 14q32.2q32.31 Microdeletion of 1.1 Mb in a 20-Year-Old Female Patient with a Maternal UPD(14)-Like Phenotype and Intellectual Disability

**DOI:** 10.1155/2014/530134

**Published:** 2014-03-30

**Authors:** Almira Zada, Farmaditya E. P. Mundhofir, Rolph Pfundt, Nico Leijsten, Willy Nillesen, Sultana M. H. Faradz, Nicole de Leeuw

**Affiliations:** ^1^Department of Human Genetics, Radboud University Medical Center, The Netherlands Division of Human Genetics, P.O. Box 9101, 6500 HB Nijmegen, The Netherlands; ^2^Center for Biomedical Research (CEBIOR), Faculty of Medicine, Diponegoro University, GSG 2nd Floor, Jl. Dr. Sutomo 14, Semarang 50244, Indonesia

## Abstract

We present a 20-year-old female patient from Indonesia with intellectual disability (ID), proportionate short stature, motor delay, feeding problems, microcephaly, facial dysmorphism, and precocious puberty who was previously screened normal for conventional karyotyping, fragile X testing, and subtelomeric MLPA analysis. Subsequent genome wide array analysis was performed on DNA from blood and revealed a 1.1 Mb deletion in 14q32.2q32.31 (chr14:100,388,343-101,506,214; hg19). Subsequent carrier testing in the parents by array showed that the deletion had occurred *de novo* in the patient and that her paternal 14q32 allele was deleted. The deleted region encompasses the *DLK1/GTL2* imprinted gene cluster which is consistent with the maternal UPD(14)-like phenotype of the patient. This rare, recurrent microdeletion was recently shown not to be mediated by low copy repeats, but by expanded TGG repeats, flanking the 14q32.2q32.21 deletion boundaries, a novel mechanism of recurrent genomic rearrangement. This is another example how the application of high resolution genome wide testing provides an accurate genetic diagnosis, thereby improving the care for patients and optimizing the counselling for family.

## 1. Introduction

The application of high resolution genome wide array analysis provides an accurate genetic diagnosis in many patients with ID and/or congenital anomalies caused by genomic imbalances. The use of this technology has led to the discovery of several novel microdeletion and microduplication syndromes. Although several patients have been reported with a terminal 14q32 deletion, patients with an in terstitial microdeletion in the 14q32 region seem to be rare. To our knowledge, only two patients with an interstitial 1.1 Mb deletion in q32.2q32.31 have previously been reported [1, 2].

Human chromosome 14q32.2 is the critical region for uniparental disomy of chromosome 14 (UPD(14)) phenotypes because it carries a cluster of imprinted genes, including the paternally expressed genes (PEGs) such as* DLK1&RTL1* and the maternally expressed genes (MEGs) such as* GTL2* (also known as* MEG3*),* RTL1as *(RTL1 antisense), and* MEG8.* Deletion of the paternal allele in this region causes a UPD(14)mat-like phenotype [3]. Uniparental disomy (UPD) occurs when the two copies of a chromosome pair are inherited from only one parent [4]. Maternal UPD of chromosome 14 (UPD(14)mat) is characterized by pre- and postnatal growth retardation, hypotonia, feeding problems, motor delay, short stature, early onset of puberty, and minor dysmorphic features of the face, hands, and feet [5]. Seven out of eleven UPD(14)mat-like cases without UPD have been reported to carry a deletion in the 14q32.2 region [2, 3, 6–9].

Here we report an additional female patient with a 1.1 Mb deletion of chromosome 14 in the q32.2q32.31 region identified by high resolution genome wide SNP array analysis.

## 2. Case Report

**Figure 1:**
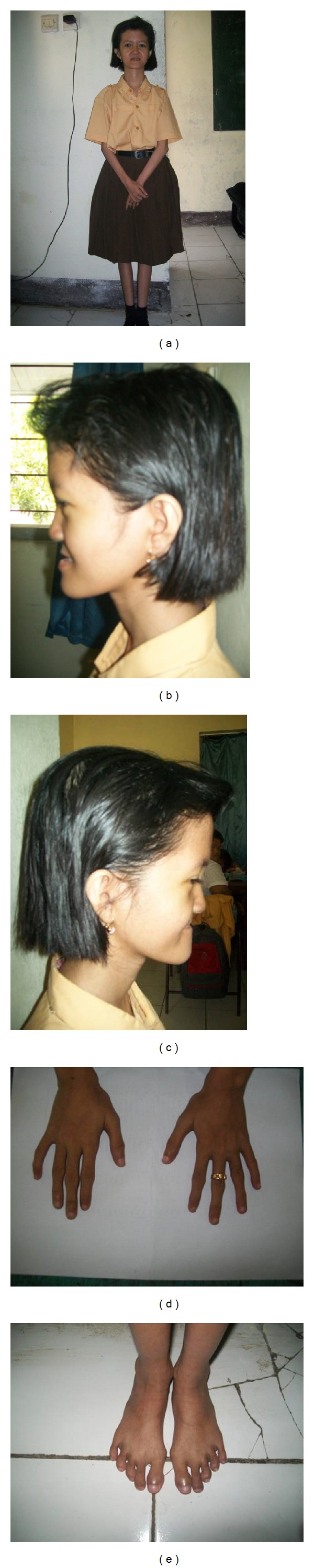
Photograph of Indonesian patient with a 14q32.2 microdeletion. Our 20-year-old patient showed extremely short and thin stature, flat face, flat philtrum, thin lips, tapering fingers, clinodactyly of the fifth finger on the right hand, and clubbing feet toes.

A 20-year-old female Indonesian Javanese patient presented with extremely thin and short stature, microcephaly, motor delay, hypotonia, mild intellectual disability, flat face, flat philtrum, thin lips, tapering fingers, clinodactyly of her fifth finger on the right hand, clubbing feet toes, feeding problems, and precocious puberty (Figure 1). She was born at 32 weeks of pregnancy with a low birth weight of 1,800 g (p5–10) as the second child of healthy, unrelated parents. At the time of her birth, her mother was 24 years of age, and her father was 33 years of age. There was no family history of intellectual disability. Postnatally, she was found to have feeding problem, motor delay, hypotonia, and precocious puberty. She was able to walk at three years of age and able to speak at two years of age. When she was examined at 20 years of age, her body height was 130 cm (*P* < 3), her weight was 18 kg (*P* < 3), and her arm span was 125 cm. Her ratio height to arm span was 0.96 cm, thus showing proportionate short stature. She has microcephaly with an occipital frontal circumference (OFC) of 50 cm (*P* < 3). Previously, genetic tests were done, including conventional karyotyping, subtelomeric MLPA, and fragile X testing, all of which showed normal results [10]. Informed consent for publishing results and photos has been obtained from the patient's parents.

**Figure 2:**
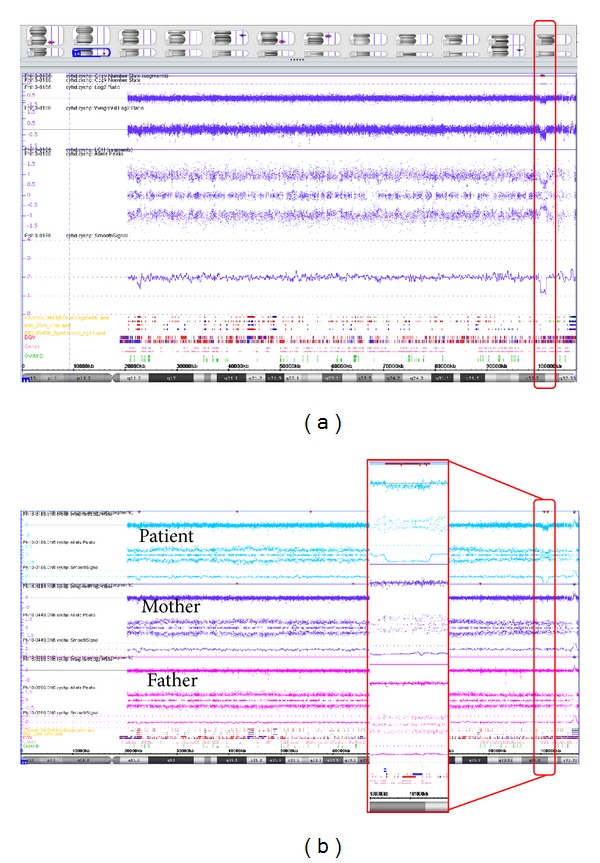
(a) Array plots of chromosome 14, visualized using the Affymetrix Chromosome Analysis Suite (ChAS) Software. A 1.1 Mb loss in 14q32.2q32.31 was detected in Patient 1 (arr[hg19] 14q32.2q32.31(100,388,343-101,506,214)x1 dn) as indicated by the red rectangle. (b) Trio analysis confirms that the deletion has occurred* de novo* in the patient.

**Figure 3:**
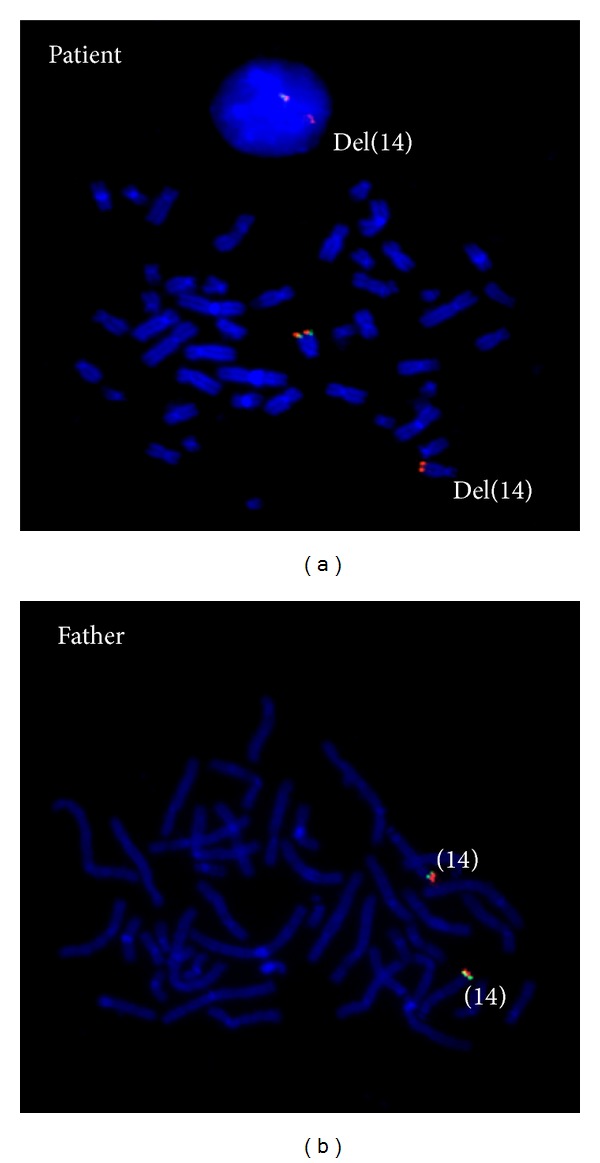
FISH study in the patient and her father. In each of them, 30 metaphases from cultured peripheral blood lymphocytes were analysed. (a) Patient metaphase showing one normal chromosome 14 [RP11-123M6 (green)/14 qter 9 (red)] and the del(14)(q32.2q32.31) with only 14 qter signal (red). (b) Metaphase from the father with a normal FISH pattern, indicating two normal chromosomes 14.

We performed genome wide array analysis on DNA from blood using the Affymetrix CytoScan HD Array platform (Affymetrix, Inc., Santa Clara, CA, USA) following the manufacturer's protocols, which showed a 1.1 Mb deletion in 14q32.2q32.31 (chr14:100,388,343-101,506,214; hg19) as depicted in Figure 2(a). Subsequent carrier testing with the same array platform in the parents revealed that the deletion had occurred* de novo* in the patient (Figure 2(b)) and that her paternal 14q32 allele was deleted. The possible presence of either a balanced chromosomal rearrangement and/or mosaic imbalance in the father was subsequently studied by fluorescence in situ hybridization (FISH) analysis using a FISH probe (RP11-123M6; BlueGnome Ltd., Cambridge, UK) specific for the 14q32.2q32.31 region. These FISH results showed that the father did not carry a balanced rearrangement and/or mosaic imbalance (Figure 3).

## 3. Discussion

Here we report an additional patient with a rare, recurrent,* de novo* 14q32.2q32.31 microdeletion of 1.1 Mb. Two other (female) patients have been reported in the literature with a similar 1.1 Mb microdeletion in 14q32.2q32.31 [1, 2]. Comparable to our patient, it concerned a* de novo* loss of paternal 14q32 allele in both patients, who exhibited clinical features compatible with UPD-(14)-mat (genomic coordinates and clinical features in hg 19 are shown in Table 1).

This deleted region comprises a snoRNA, part of a microRNA cluster, and 15 protein-coding genes, containing a cluster of imprinted genes including paternally expressed genes* DLK1&RTL1* and maternally expressed genes* GTL2* (also known as* MEG3*),* RTL1as,* and* MEG8*. The deleted paternal 14q32 allele causes a maternal UPD(14)-like phenotype [3]. The other 10 genes are not imprinted (*EVL, DEGS2, YY1, SLC25A29, c14orf68, WARS, WDR25, BEGAIN, c14orf70,* and the 3'end of* EML1*).

Other than pre- and postnatal growth retardation, precocious puberty, and feeding problems, which are compatible with UPD(14)mat phenotypes, the patient reported here also manifested intellectual disability (ID) which is not or rarely observed in patients with UPD(14)mat. Considering that the 1.1 Mb 14q32 deletion also encompasses many nonimprinted genes, it is likely that the ID in this patient is caused by haploinsufficiency of one or more dosage-sensitive genes. For example, the* BEGAIN* (brain-enriched guanylate kinase-associated protein) gene represents a good candidate based on its localization in the neuronal synapse [11]. The recently reported* YY1* gene is considered to be an ID disease gene based on the exome sequencing in a patient with unexplained ID in whom a missense mutation c.1138G>T with protein level change p.Asp380Tyr was found [12]. This gene implicates chromatin remodelling as its main function by encoding the ubiquitously expressed transcription factor yin-yang 1 and directing histone acetylases and histone acetyltransferases. Experiments with Yy1 knockdown mice resulted in growth retardation, neurulation defects, and brain abnormalities [13].

Most recurrent genomic rearrangements are due to nonallelic homologous recombination (NAHR) between misaligned low copy repeats resulting in a microdeletion or a microduplication [14]. However, a recent paper by Béna et al. [1] reported the observations regarding the mechanism underlying the recurrent 14q32.2 deletion described here. They found that large (TGG)n tandem repeat tracts of about 500 bp are at both boundaries of the deletion (chr14:100,394,091-100,394,594 and chr14:101,504,592-101,505,016; hg19). The TGG repeats are the longest type of triplets motif and highly capable of forming G4 DNA. Some theories might explain how these triplet repeats can cause genomic rearrangement. First, expanded triplet repeats would provide an aggravated substrate for genomic rearrangement through the NAHR mechanism [15, 16]. Second, expanded triplet repeats have the tendency to form intramolecular secondary structures termed guanine quadruplexes or G4 DNA which could promote double strand chromosome breaks [17]. Therefore, it is suggested that this recurrent 14q32.2 microdeletion is mediated by expanded TGG repeats, a novel mechanism of recurrent genomic rearrangement that is shown not to be mediated by low copy repeats.

In conclusion, we were able to detect a rarely identified 14q32.2 microdeletion by using high resolution genome wide array analysis in a patient whose genotype and phenotype are in agreement with those of two previously reported patients in the literature. This case report demonstrates the value of applying high resolution genome wide testing for accurate genetic diagnosis that can help to improve the care for patients and to optimize the counselling for family.

## Figures and Tables

**Table 1:** Genomic coordinates and clinical features of all cases.

	Buiting et al., 2008 [2]	Béna et al., 2010 [1]	Present case
Deletion positions (Mb) in chromosome 14 (hg 19)	100.396–101.502	100.400–101.500	100.388–101.506
Sex	Female	Female	Female
Age (years)	14 $\frac{1}{4}$	4	20
Pre- and postnatal growth retardation	+	+	+
Hypotonia	+	+	+
Feeding problems	+	+	+
Precocious puberty	+	?	+
Intellectual disability	+	+	+
Dysmorphism	−	High forehead, small chin, posteriorly rotated ears, and flat feet	Flat face, flat philtrum, thin lips, tapering fingers, clinodactyly of the fifth finger on the right hand, and clubbing feet toes
Others	−	Hypermetropia	−

+: present; −: not present; ?: undetermined yet.
